# Analgesia nociception index as a tool to assess the effectiveness of paravertebral block in total mastectomy: a prospective cohort study

**DOI:** 10.1016/j.bjane.2025.844699

**Published:** 2025-11-07

**Authors:** Julien Raft, Anne-Sophie Lamotte, Cécile Huin Schohn, Caroline Fritz, Philippe Richebé

**Affiliations:** a Institut de Cancérologie de Lorraine, Department of Anesthesiology, Vandoeuvre-lès-Nancy, France; b University of Lorraine, INSERM DCAC, Vandoeuvre-lès-Nancy, France; c University of Lorraine, CRAN, Vandoeuvre-lès-Nancy, France; d Institut de Cancérologie de Lorraine, Department of Clinical Research, Vandoeuvre-lès-Nancy, France; e Annecy Genevois Hospital, Department of Anesthesiology, Annecy, France; f Polyclinique Bordeaux Nord Aquitaine, Department of Anesthesiology, Bordeaux, France; g University of Montreal, Maisonneuve-Rosemont Hospital, Department of Anesthesiology and Pain Medicine, Montréal, QC, Canada

**Keywords:** Analgesia, Mastectomy, Pain, Pain measurement, Regional anesthesia

## Abstract

**Background:**

Single-injection Paravertebral Block (PVB) is commonly used for analgesia in major breast surgery; however, its sensory effectiveness may be variable. This study investigated whether intraoperative changes in the Analgesia Nociception Index (ANI) are associated with PVB effectiveness.

**Methods:**

This prospective observational study included 100 women scheduled for total mastectomy. A single-injection PVB was performed preoperatively under ultrasound guidance at the T3 level. Sensory testing was performed from T1 to T10, but block effectiveness was evaluated in the surgical field (T2–T6). PVBs were classified as effective (complete loss of cold sensation in all T2‒T6 dermatomes) or incomplete (partial cold sensation in this range). ANI variations, intraoperative remifentanil consumption, postoperative pain scores, and morphine use were compared.

**Results:**

Ninety-three patients were analyzed. PVB was effective in 75% and incomplete in 25%. The mean ANI variation was significantly greater in the effective group (+1.4 ± 10.3) compared to the incomplete group (-11.0 ± 7.1), with a mean difference of 12.4 (95% CI: 8.8 to 16.0; p < 0.0001). Remifentanil consumption was higher in the incomplete group (0.072 ± 0.018 µg.kg^−1^.min^−1^ vs. 0.054 ± 0.008 µg.kg^−1^.min^−1^), mean difference 0.018 (95% CI: 0.010 to 0.026; p < 0.0001). Pain score and morphine consumption were significantly higher for patients with incomplete PVB.

**Conclusion:**

In this observational study, a significant decrease in ANI values following skin dissection was associated with incomplete PVB. Early ANI monitoring may help identify insufficient regional block during total mastectomy, thus guiding intraoperative analgesic adjustment to improve patient comfort.

## Introduction

Paravertebral Block (PVB) is a regional anesthesia technique in which a Local Anesthetic (LA) is injected into the thoracic paravertebral space between the costovertebral ligament and the pleura.^1^^,^^2^ Ultrasound guidance has improved the safety and efficacy of this block, allowing a single injection with a larger volume, now commonly used for major breast surgery.^3^ The breast is primarily innervated by thoracic dermatomes T2 to T6, and axillary dissection requires T2 coverage. However, the metameric spread of single-level PVB remains unpredictable, as demonstrated by an imaging study.^4^

PVB is usually performed just before induction or under General Anesthesia (GA), but its efficacy is difficult to assess intraoperatively. Traditional hemodynamic parameters such as blood pressure or heart rate are insufficiently sensitive to detect nociceptive responses reliably.^5^, ^6^, ^7^ This makes real-time detection of incomplete blocks challenging. The Analgesia Nociception Index (ANI) (Metrodoloris Medical Systems, Lille, France) was developed to monitor the balance between nociception and antinociception during GA. It is based on heart rate variability analysis and reflects parasympathetic nervous system tone. ANI values tend to decrease in response to nociceptive stimuli and remain stable when analgesia is adequate.^8^^,^^9^ Several studies have shown promising results for ANI as a nociceptive monitor during general anesthesia.

We hypothesized that variations in ANI following skin dissection could reflect the efficacy of preoperative PVB in patients undergoing total mastectomy under general anesthesia. The objective of this prospective observational study was to assess whether a decrease in ANI values shortly after skin incision is associated with insufficient regional analgesia.

## Methods

### Study setting

This prospective observational study was conducted at the Cancer Center (Institut de Cancérologie de Lorraine, Nancy, France) from May 2, 2019, to June 26, 2020. The objective was to determine whether a decrease in the mean ANI (ANIm) 1 minute after the end of the initial skin dissection could be associated with an incomplete or ineffective PVB. An incomplete or ineffective PVB was defined by the presence of cold sensation during an ice cube test between thoracic metameric levels T2 and T6. This study was approved by the French National Ethics Committee of the SUD-EST IV (Approval n° ID-RCB: 2019-A00121-56) and registered in ClinicalTrials.gov (NCT03832920). Informed consent was obtained from all participants.

The sample size was determined a priori using PASS software (version 08.0.15, NCSS, USA). Based on previous literature, we assumed a 10% incidence of incomplete or ineffective PVB.^4^, ^10^, ^11^ Detecting this failure rate with a two-sided α risk of 5% and a statistical power of 80% (β = 0.20) required 100 patients. This calculation was based on a one-sample proportion test against the null hypothesis of a negligible failure rate (< 2%). The target sample size was also consistent with previous studies evaluating regional anesthesia efficacy in breast surgery.

### Study participants

We enrolled 100 women aged 18 to 85 years with an American Society of Anesthesiologists (ASA) Physical Status of 1‒3 and a body mass index between 17 and 30 kg.m^−2^, scheduled for total mastectomy with or without sentinel lymph node dissection.

Exclusion criteria included: male sex, any interaction with physiological sinus rhythm (chronic arrhythmias, pacemaker, heart transplantation), any treatment affecting parasympathetic or sympathetic tone (e.g., beta blockers, intraoperative atropine administration), diabetes, neuromuscular disease, pregnancy, breastfeeding, bilateral surgery, chronic pain, LA allergy, infection at puncture site, immediate breast reconstruction, or protocol non-compliance.

### Study protocol

No premedication was administered. An intravenous catheter was inserted into the forearm or hand for medication delivery. In the preoperative holding area of the Post-Anesthesia Care Unit (PACU), standard monitoring including electrocardiography, pulse oximetry, and non-invasive blood pressure monitoring was placed. Patients were positioned in the lateral decubitus position on the contralateral side of the surgery. The third thoracic paravertebral space (T3) was scanned using ultrasound (Model Sonosite SII, Fujifilm, Paris, France) with a 15‒6 MHz linear probe. The T3 paravertebral level was identified using an ultrasound-guided anatomical counting method. The transducer was placed in a parasagittal orientation starting at the first rib, and ribs were counted caudally to locate the third rib. Then, the probe was placed in the transverse plane against the spinal process. Under aseptic conditions, a 22-gauge, 80 mm needle (SonoTAP, Pajunk, Germany) was advanced in an in-plane direction toward the paravertebral space, positioned immediately above the pleura and below the costotransverse ligament. The needle’s position was confirmed by observing the descent of the pleura upon injecting 2‒3 mL of saline for hydrolocalization. Subsequently, 20 mL of 7.5 mg.mL^−1^ ropivacaine was injected, with intermittent negative aspiration tests conducted every 5 mL. All paravertebral blocks were performed by senior anesthesiologists with over five years of experience in regional anesthesia in breast surgery and proficiency in ultrasound-guided thoracic blocks (more than 100 PVB performed). This consistency in operator experience aimed to reduce variability in block performance and ensure a reproducible technique across all patients.

Patients were transferred to the operating room no sooner than 15 minutes later. Just before GA induction, a thin ice block was used to conduct the cold sensation test on the anterior chest (results of the test blinded to the rest of the team). Patients were provided with a reference cold sensation on a thigh prior to measurement. The peak sensory cephalad and caudal block levels were assessed, and the number of blocked dermatomes was recorded. Routine monitoring was conducted in accordance with French guidelines. Intraoperative monitoring included the Bispectral Index (BIS) (Medtronic, Paris, France) and the Analgesia/Nociception Index (ANI, MDoloris Medical Systems, France). The ANI is a 0‒100 index, with higher values (above 50) indicating a predominant parasympathetic tone (comfort, analgesia, adequate nociception/antinociception balance), while lower values (below 50) suggest a predominant sympathetic tone (stress, pain, inadequate nociception/antinociception balance). Dynamic variations in ANI provide better predictive performance for hemodynamic reactivity during GA than static values.^9^ The ANI monitor continuously displays the instant ANI (ANIi), calculated every second, and the mean ANI (ANIm), which reflects the average ANI over the previous three minutes. In this study, ANIm was selected as the primary outcome measure because it provides a more stable and reliable indicator of autonomic balance during general anesthesia, by smoothing out transient fluctuations unrelated to nociceptive events. This makes it particularly suitable for assessing the nociceptive response to a defined surgical stimulus such as skin dissection. In the event of block failure (e.g., persistent cold sensation in all dermatomes T2‒T6), intraoperative analgesia was managed using increased remifentanil infusion and, if necessary, rescue boluses of morphine in the PACU.

Anesthesia was induced and maintained with intravenous propofol targeting an effect concentration according to the BIS index (40‒60) and intravenous remifentanil targeting an effect-site concentration based on the ANI index (over 60) and any nociceptive hemodynamic responses detected by the anesthesiologist. The choice of neuromuscular blocking agent, airway management, and lung ventilation strategies were left to the discretion of the anesthesiologist. Antiemetic prophylaxis and postoperative pain management included an intravenous injection of 8 mg dexamethasone at induction and paracetamol (1,000 mg) and ketoprofen (1 mg.kg^−1^) administered at the end of the mastectomy before skin closure. The laryngeal mask or tracheal tube was removed in the operating room after reversal of neuromuscular blockade, when needed, and patients were then transferred to the PACU.

Postoperative pain intensity at rest was assessed upon arrival in the PACU and every 30 minutes using a Visual Analog Scale (VAS) ranging from 0 (no pain) to 10 (worst imaginable pain). If a VAS score exceeded 3/10 at rest in the PACU, intravenous morphine was titrated using 1 mg boluses every 5 minutes (with no limit in the dosage). Patients remained in the PACU until the Aldrete score was above 9/10 and the VAS score was less than or equal to 3. If nausea or vomiting occurred, 4 mg IV ondansetron was administered, followed by 1.25 mg IV droleptan if symptoms were insufficiently controlled.

### Data collection

All study data were securely recorded and managed using CleanWeb (Telemedicine Technologies S.A.S.). For each patient meeting the inclusion criteria, the following parameters were measured: ANIm before surgical skin incision without stimulation, ANIm 1 minute after the end of the breast surgical skin dissection cephalad and caudal block level limits, any intraoperative administration of IV atropine or IV ephedrine, pain scores using a VAS at arrival and discharge from the PACU, and ultrasound visualization of the paravertebral space (good or bad).

**Fig 1:**
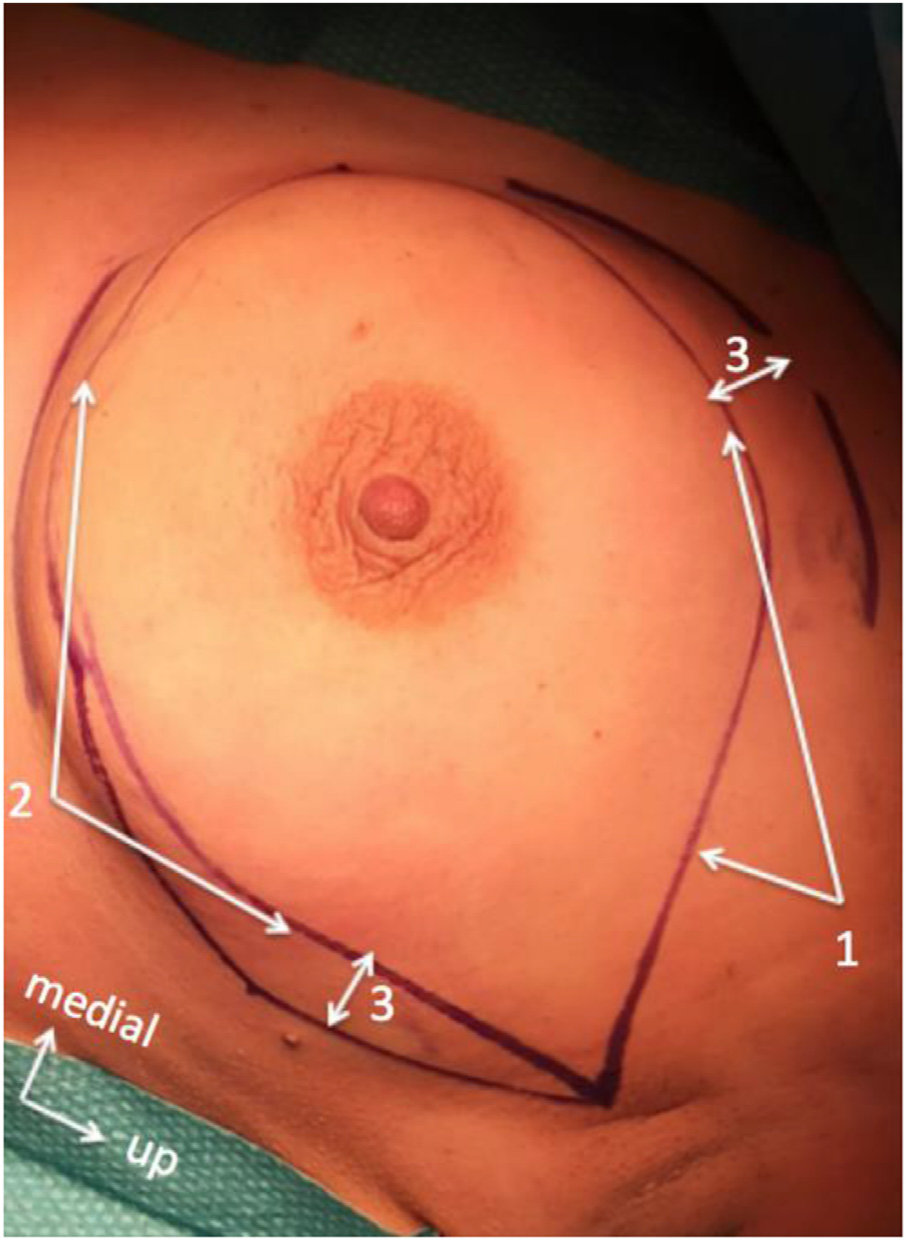
Surgical lines for breast skin dissection (1 = Line for upper total mastectomy incision, 2 = Line for lower total mastectomy incision, 3 = Minimal skin conservation for closure).

The skin dissection included the upper and lower skin incisions (Fig. 1), hemostasis, and the separation of skin from the mammary gland before deep tissue dissections.

Sensor level of efficacy was assessed using an ice cube test, which allowed classifying blocks as effective, incomplete or ineffective. A standardized cold sensation test using an ice cube was performed by a blinded evaluator to assess the presence or absence of sensory block in the thoracic dermatomes corresponding to the surgical field. The test was applied bilaterally on the anterior and lateral chest wall, typically from T1 to T10, using a non-standardized cranio-caudal sequence. The presence of cold sensation between T2 and T6 was used to define an incomplete or ineffective block. Although testing was extended from T1 to T10 for completeness, only the T2–T6 dermatomes, corresponding to the mastectomy surgical field, were considered for block efficacy assessment. Dermatomes of the upper limb were not assessed, as the focus was on regional anesthesia coverage of the breast and chest wall. The ice cube sensation test defined four groups: an effective group (no sensation between thoracic levels T2 and T6), an incomplete group (no sensation at 1, 2, 3, or 4 levels between T2 and T6), an ineffective group (no blocked levels), and a failure group (the sum of the incomplete and ineffective groups). The evaluation was systematically performed by the same trained anesthesiologist to ensure consistency and reduce inter-observer variability.

### Outcomes

The primary outcome was the comparison of the variation in ANIm (before skin incision and 1 minute after the end of the surgical skin dissection) among the different groups (effective, incomplete, and ineffective). For the analyses, the ANI change (ΔANI) was calculated as the post-dissection ANIm value minus the pre-incision ANIm value.

The secondary outcomes included comparisons between PVB effectiveness groups for the following variables: intraoperative remifentanil consumption, PACU morphine consumption, and pain at rest in the PACU upon arrival and before discharge.

### Statistical analysis

In univariate descriptive analysis, qualitative parameters were described by frequency and percentage, while quantitative parameters were described by mean ± standard deviation, median, minimum – maximum, and 1^st^ and 3^rd^ uartiles. In bivariate analysis, qualitative parameters were compared using a Chi-Squared test. Quantitative parameters were compared using the parametric Student's *t*-test if normality was met (Shapiro-Wilk test), or the non-parametric Wilcoxon-Mann-Whitney test, otherwise.

Effect sizes (mean differences) were calculated for continuous variables and corresponding 95% Confidence Intervals (95% CI) were reported to quantify the magnitude and precision of differences between groups. For categorical outcomes, Odds Ratios (OR) with 95% CIs were computed.

To evaluate the ability of ANIm values to predict effective PVB, a Receiver Operating Characteristic (ROC) curve analysis was performed. The area under the ROC Curve (AUC) was calculated to assess diagnostic performance, and the optimal threshold was defined using the Youden Index.

No multivariable regression analysis was performed because of the limited number of events (23 incomplete blocks) relative to the sample size, which would not allow for a robust model without risking overfitting. In addition, the study population was deliberately homogeneous (ASA I–III women, standardized oncologic breast surgery, and a uniform anesthesia protocol), which reduced the likelihood of major confounders. Nevertheless, we acknowledge this absence of multivariable adjustment as a limitation, as residual confounding cannot be fully excluded. However, this limitation is acknowledged in the discussion. No missing data were recorded for primary or secondary outcome variables (ANI values, pain scores, or opioid consumption) in the 93 analyzed patients.

The significance level was set at 5%. All analyses were conducted using RStudio software (version 2022.07.2+576; RStudio, Inc., Boston, USA).

## Results

### Demographics

**Fig 2:**
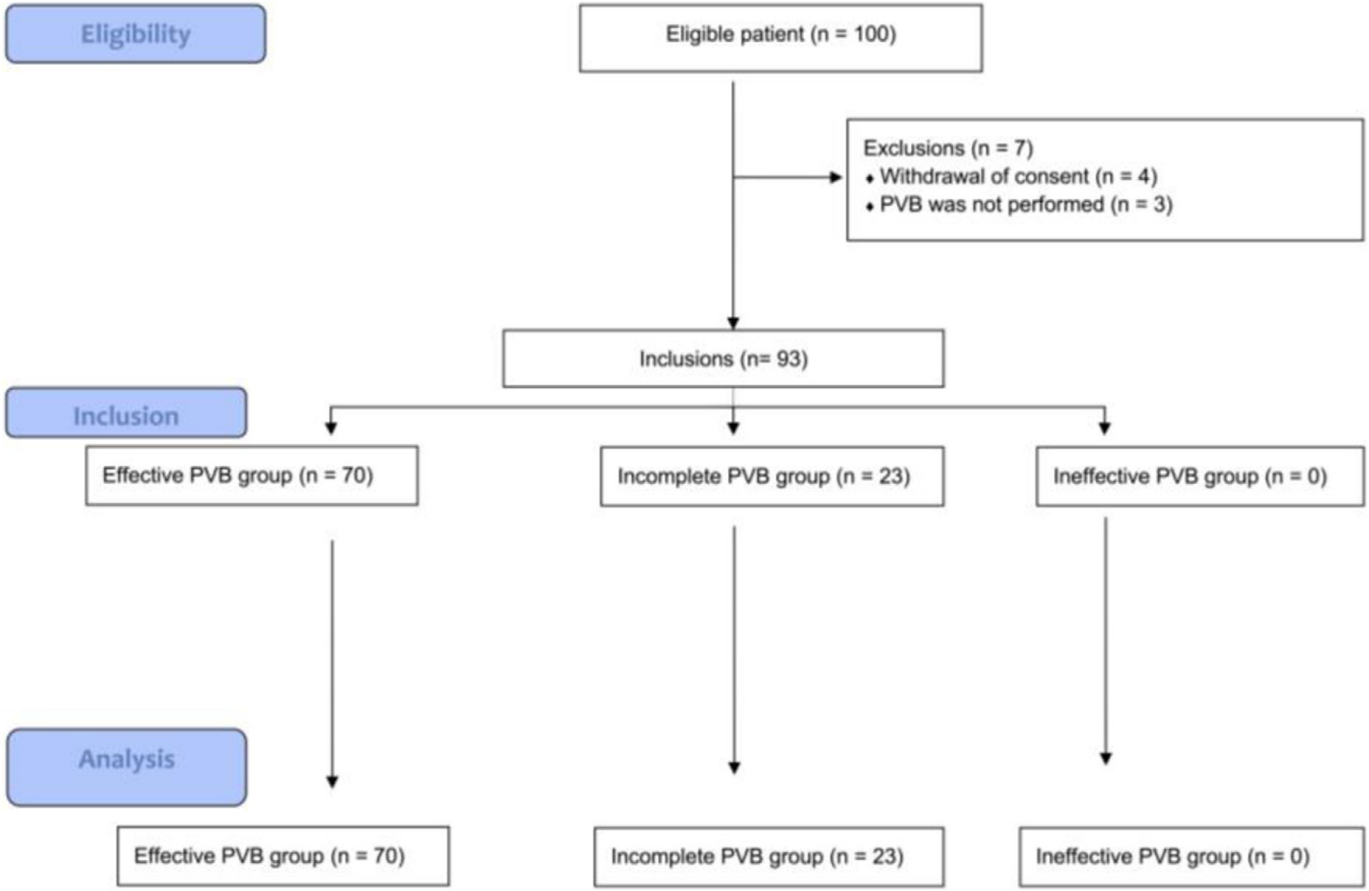
Flowchart (PVB, Paravertebral Block).

One hundred women were screened and included in this study. Ninety-three patients were analyzed, as seven were excluded (four due to withdrawal of consent and three because PVB was not performed) (Fig. 2).

The demographic data of the patients are as follows: the mean age was 60.4 ± 12.8 years, and the mean Body Mass Index (BMI) was 24.3 ± 4.0 kg.m^−2^. The distribution of the American Society of Anesthesiologists (ASA) Physical Status was as follows: 37% ASA I, 60% ASA II, and 3% ASA III. The proportion of patients undergoing sentinel lymph node dissection was 30% with no difference in outcomes compared to those underdoing total mastectomy without axillary surgery. Ultrasound visualization of the paravertebral space was deemed good in 96% of cases and poor in 4%. The mean duration of surgery was 67 ± 24 minutes, and the mean duration in the PACU was 77 ± 26 minutes. Demographic and baseline characteristics per group were reported in Table 1.

**Table 1:** Demographic and clinical data. Data are presented as mean ± SD or percent. In bivariate analysis, qualitative parameters were compared using a Chi-Squared test. Quantitative parameters were compared using the parametric Student's *t*-test if normality was met (Shapiro-Wilk test), or the non-parametric Wilcoxon-Mann-Whitney test otherwise.

	Incomplete PVB (n = 23)	Effective PVB (n = 70)	p-value
**Age (years)**	57.4 ± 12.7	61.3 ± 12.7	0.2
**Body Mass Index**	24.43 ± 4.95	24.28 ± 3.66	0.9
**ASA I/II/III (%)**	44/52/4	34/63/3	0.47
**Type of surgery (%)**			
Total mastectomy alone	65	71	0.76
Total mastectomy and sentinel lymph node dissection	35	29	
**Good ultrasound visualization (%)**	95	97	1
**Duration of** s**urgery (min)**	64 ± 19	68 ± 25	0.46

PVB, Paravertebral Block.

The distribution of results from the cold sensation test was as follows: effective group 75% (n = 70), incomplete group 25% (n = 23), and ineffective group 0%. Although sensory testing was systematically performed from T1 to T10 on the anterior chest wall, the classification of block efficacy was based on the presence or absence of cold sensation between T2 and T6, corresponding to the mastectomy surgical field. The upper metameric extensions were T1 15%, T2 62%, T3 16%, and T4 7%. The lower metameric extensions were T3 2%, T5 6%, T6 24%, T7 28%, T8 24%, T9 10%, and T10 6%. No intraoperative administration of IV atropine or IV ephedrine was reported. Since thoracic PVB does not affect the brachial plexus, no motor blockade of the upper limb is expected. Consequently, no formal motor assessment of the upper extremity was performed, and no motor symptoms were reported by patients.

### Outcomes

**Fig 3:**
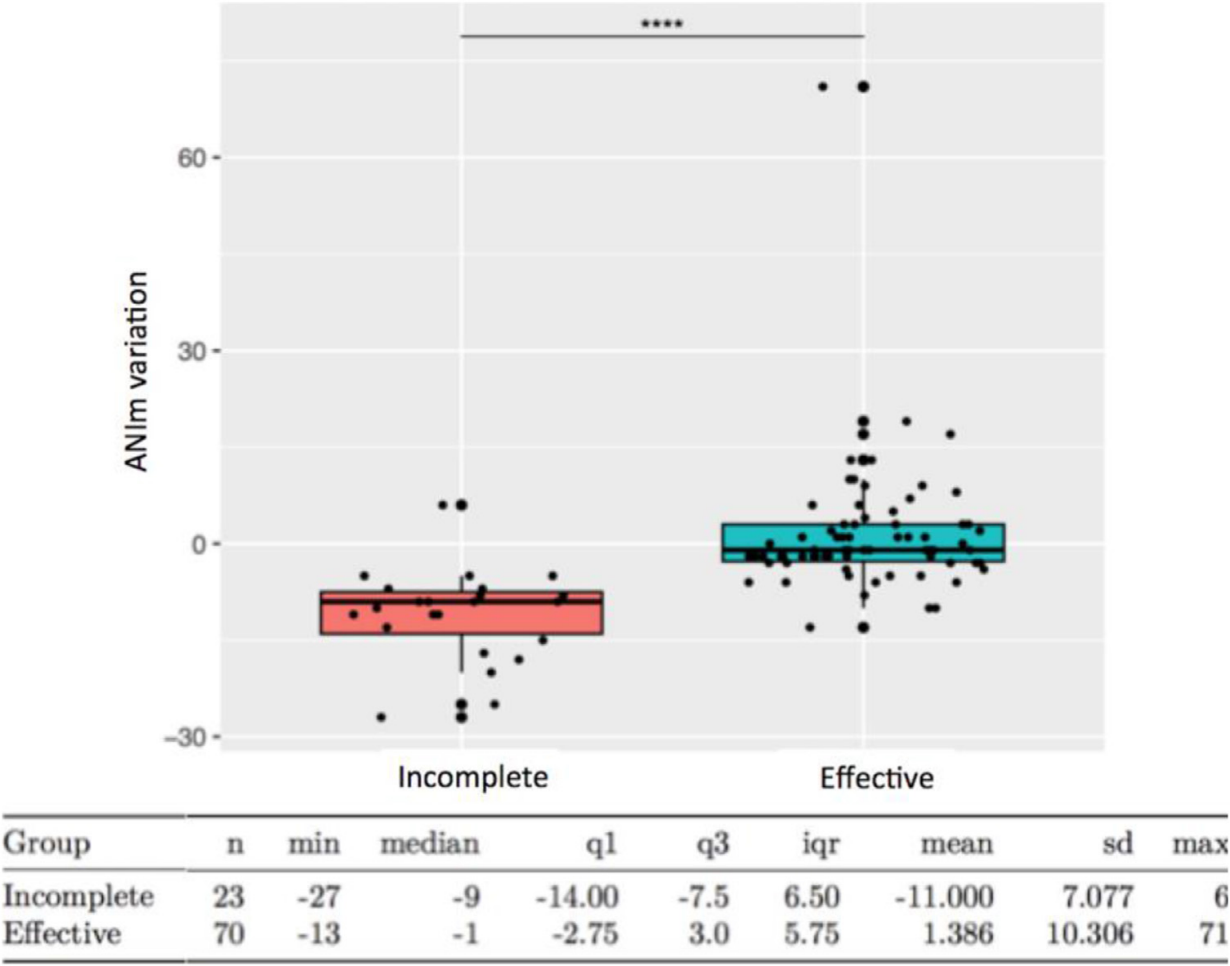
ANIm variation from before surgical skin incision to one minute after the end of skin dissection (Wilcoxon test p < 0.0001) (min, Minimal; q1, 1^st^ quartile; q3, 3^rd^ quartile; iqr, Interquartile range; sd, Standard Deviation; max, Maximal).

The mean variation of ANIm, from before the surgical incision to one minute after the end of the skin dissection, decreased significantly in the incomplete PVB group -11.0 ± 7.1 compared to the effective PVB group 1.4 ± 10.3, with p < 0.0001 (Fig. 3). While the absolute difference in ANIm variation may seem modest, its clinical relevance is evident from consistent differences in intraoperative remifentanil consumption, postoperative pain scores, and PACU morphine use. Moreover, the ROC analysis demonstrated good predictive performance, indicating that even relatively small changes in ANIm values may reflect meaningful differences in analgesic efficacy.

**Fig 4:**
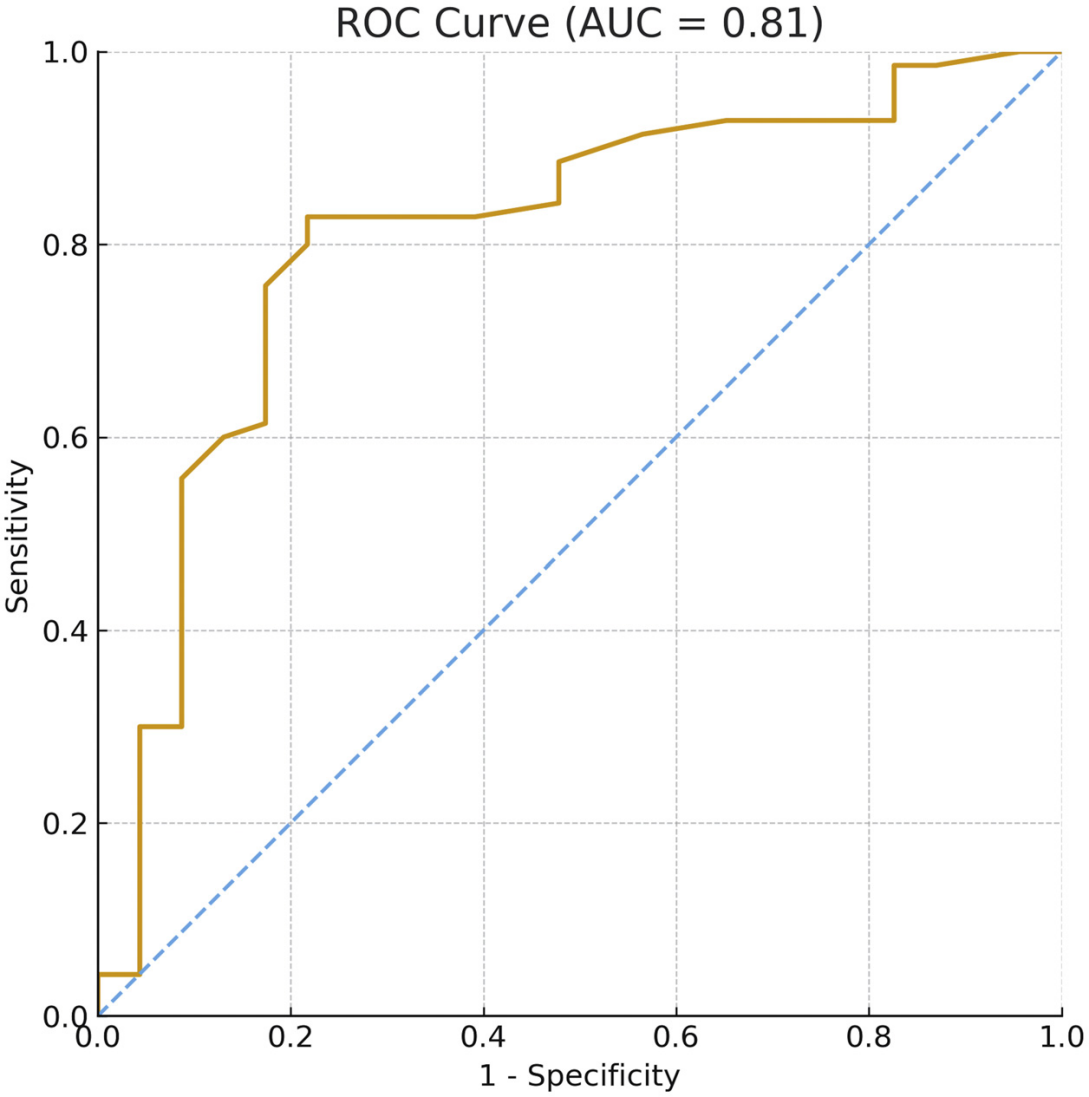
Receiver Operating Characteristic (ROC) curve assessing the predictive performance of ANIm.1 min for identifying effective Paravertebral Blocks. The Area Under the Curve (AUC) was 0.810, indicating good discriminative ability. A cutoff value of 63.5 for ANIm.1 min was identified using the Youden Index as the optimal threshold, providing the best balance between sensitivity and specificity in predicting effective block coverage from T2 to T6.

The area under the ROC Curve (AUC) was 0.81, indicating good discriminative power for differentiating between effective and incomplete blocks (Fig. 4). The optimal threshold was identified using the Youden Index, corresponding to an ANIm.1 min (one minute after the end of skin dissection) score of 63.5. Patients with ANIm.1 min ≥ 63.5 were 17.4 times more likely to present with an effective block (OR = 17.4; 95% CI: 5.76–61.7; p < 0.001).

**Fig 5:**
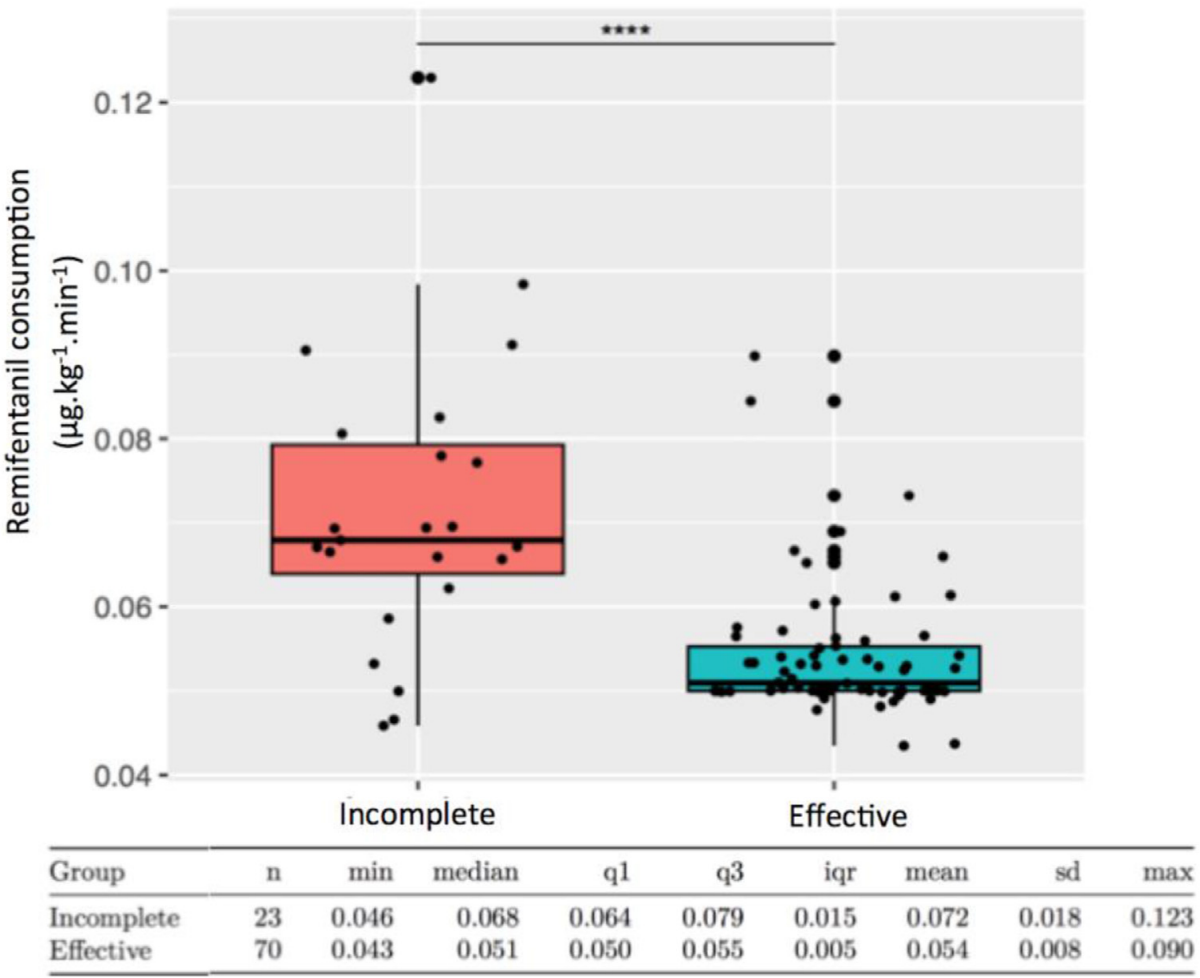
Intraoperative consumption of remifentanil (Wilcoxon test p < 0.0001) (min, Minimal; q1, 1^st^ quartile; q3, 3^rd^ quartile; iqr, Interquartile range; sd, Standard deviation; max, Maximal).

The mean intraoperative consumption of remifentanil was significantly higher in the incomplete group 0.072 ± 0.018 µg.kg^−1^.min^−1^ compared to the effective group 0.054 ± 0.008 µg.kg^−1^.min^−1^, p < 0.0001. The mean difference was 0.018 µg.kg^−1^.min^−1^ (95% CI: 0.010 to 0.026) (Fig. 5).

The mean pain scores in the PACU were significantly higher in the incomplete group: upon arrival, the incomplete group had a score of 3.7 ± 2.4 vs. 0.7 ± 1.1 in the effective group, mean difference 3.0 (95% CI: 1.94 to 4.07) p < 0.0001, and before discharge the PACU, the incomplete group scored 2.1 ± 0.9 vs. 1.0 ± 1.1 in the effective group, mean difference 1.1 (95% CI: 0.64 to 1.56) p < 0.0001. The mean morphine consumption during the PACU was significantly higher in the incomplete group 1.8 ± 1.5 mg compared to the effective group 0 ± 0.2 mg, the mean difference was 1.8 mg (95% CI: 1.15 to 2.45) p < 0.0001.

## Discussion

This study highlights the relationship between ANI variations and the sensory effectiveness of PVB for total mastectomy. ANI measurements can also serve as a valuable tool directly at the beginning of surgery to assess the level of analgesia provided by the PVB. This study confirms the interindividual variability in sensory blockade achieved through single PVB injection.^4^ Our data show a clear variation between effective and incomplete PVB distribution levels, also with a significant decrease in ANIm values reported after skin dissection.

Given the primary objective of evaluating the association between ANI variation and block efficacy, secondary outcomes (analgesic consumption, pain scores) were considered exploratory. Therefore, no correction for multiple comparisons was applied, as the analysis was not intended for definitive inferential testing but rather hypothesis generation. However, we acknowledge this as a limitation and interpret secondary findings with appropriate caution.

This significant variation of ANI supports the notion that ANI can accurately represent patients' nociceptive state directly at the beginning of the surgery. Although no standardized threshold for ANI change has been defined, several studies have shown that dynamic variations, particularly sudden decreases in ANI in response to nociceptive stimuli, correlate with inadequate analgesia.^12^^,^^13^ Our findings are consistent with this interpretation, as patients with incomplete blocks showed a significant decline in ANI following skin dissection. These changes may be more clinically meaningful than absolute values. ANI is effective not only in detecting but also in predicting an inadequate balance between nociception and antinociception during GA. However, variations in ANI may also be influenced by factors such as hemodynamic fluctuations, residual autonomic tone, or insufficiently stable anesthetic depth. Although propofol and remifentanil infusions were titrated using BIS and ANI targets, we acknowledge that intraoperative fluctuations could have introduced variability.

While the exact time between PVB completion and surgical incision was not recorded, all blocks were followed by a mandatory 15 minutes interval before sensory assessment, with general anesthesia and surgical preparation occurring thereafter. Given the pharmacodynamic profile of ropivacaine at 7.5 mg.mL^−1^, typically achieving onset within 10 to 15 minutes, we believe that the LA had sufficient time to take effect prior to incision in all patients, even though the precise interval to incision was not recorded.^14^^,^^15^ This delay, combined with block performed before general anesthesia, supports the reliability of sensory evaluation.

The lateral pectoral nerve is described as receiving nerve fibers from C5 to C7 nerve roots and the medial pectoral nerve from C8 and T1 with some variations.^16^ They are primarily involved in motor innervation of the pectoral muscles, with only limited or indirect contribution to sensory perception in the anterior chest wall. These considerations could influence pain management during and after surgery. In our study, the maximal upper metameric extension was T1. PVB does not adequately cover the sensory distribution of these nerves, patients may experience pain during surgery, particularly during the dissection and manipulation of the pectoral muscles. Thus, early assessment during skin dissection may not fully capture incomplete regional analgesia across the entire breast. However, the lower mean intraoperative remifentanil consumption in the effective group of this study suggests that this influence is weak.

No adverse events or signs of Local Anesthetic Systemic Toxicity (LAST) were observed in this study. In this study, a fixed dose of 20 mL of 7.5 mg.mL^−1^ ropivacaine (150 mg) was administered for all blocks, which remained well below the recommended maximum dose of 3 mg.kg^−1^ or 200 mg.^17^ Although the dose was not adjusted to body weight, no adverse effects were observed, and the fixed dosing protocol reflects standard practice in our institution. This is standard clinical practice for breast surgery. Ropivacaine is associated with a favorable safety profile due to its reduced cardiotoxicity and central nervous system toxicity compared to bupivacaine. Nevertheless, practitioners should remain vigilant for signs of LAST, particularly when using higher concentrations or in patients with low body mass or altered metabolism. Ultrasound guidance and aspiration before injection were systematically used to reduce the risk of intravascular administration. The absence of complications in our cohort further supports the safety of this approach when appropriately performed.

### Strengths and limitations

The main strengths of this study include its prospective design, the homogeneous population of ASA I–III women undergoing standardized oncologic breast surgery, and the use of a uniform anesthetic protocol. The absence of missing data for primary and secondary outcomes and the blinded sensory assessment before anesthesia induction further reinforce the internal validity of the findings.

Nevertheless, several limitations must be acknowledged. First, the study was observational and lacked randomization, which limits causal inference. Another limitation is the absence of multivariable adjustment. Although our study design and inclusion criteria aimed to minimize heterogeneity and potential confounding, residual confounders such as BMI, anxiety, or age-related autonomic variability may still have influenced ANI values. The relatively small number of incomplete blocks also precluded a meaningful multivariable analysis. Future larger studies should address this point with adequate adjustment. Furthermore, although BMI was recorded, no subgroup analysis was performed due to the limited sample size. Preoperative anxiety was not assessed, which may have influenced postoperative pain scores, representing an additional uncontrolled confounding factor.

Another limitation is the potential for incorporation (circularity) bias, as ANI values guided intraoperative remifentanil titration and were also used as a predictive variable. This dual role may have partially influenced the observed associations, although the consistency across analgesic outcomes (remifentanil consumption, PACU pain scores, and morphine use) supports the robustness of our findings.

Although the ice cube test is inherently subjective, its implementation in our study was designed to minimize potential bias. In particular, the assessment of the sensory block was conducted by a dedicated evaluator who was blinded to all intraoperative and postoperative outcomes. This evaluator performed the test just prior to the induction of general anesthesia and was not involved in the performance of the PVB or subsequent anesthesia management. Conversely, the anesthesiologists who managed intraoperative care and collected hemodynamic and analgesic data were not informed of the results of the sensory evaluation. This structure ensured a partial blinding model, in which the team performing the block and anesthesia did not influence nor were influenced by the sensory assessment outcome. Participants were not blinded to the sensory block assessment, which may have introduced bias in subjective pain reporting during the postoperative period. Although full double-blinding was not feasible in this observational setting, this separation of roles helped reduce potential observer and performance bias. However, interindividual variability in cold perception, the lack of inter-rater reliability assessment, and reliance on a single sensory modality (cold) may have introduced classification bias. The ice cube test does not assess other relevant sensory modalities (e.g., mechanical or nociceptive), which could affect the accuracy of block effectiveness classification.

Finally, this study did not include a stratified analysis of ANI responses or block effectiveness based on age. Moreover, the external validity of our findings may be limited due to the single-center nature of the study and the homogeneous population of ASA I–III women undergoing standardized oncologic breast surgery. These findings may not be generalizable to more diverse patient populations or clinical settings. Given the known physiological decline in autonomic responsiveness and heart rate variability in older adults, age may influence ANI measurements.^18^ Further studies are needed to assess whether ANI thresholds should be adjusted according to patient age in the context of regional anesthesia.

## Conclusion

In conclusion, this observational study suggests that intraoperative variations in ANI values may help identify ineffective paravertebral blocks during breast surgery. Patients with incomplete blocks demonstrated significant ANI decreases after skin dissection, alongside higher opioid requirements and postoperative pain scores. These findings support the potential role of ANI monitoring as an adjunctive tool for the early detection of inadequate regional analgesia. However, due to the non-randomized design of the study, no causal inference can be made. Future randomized controlled trials are needed to confirm whether ANI-guided intraoperative analgesia management improves clinical outcomes. Based on our ROC analysis, an ANIm threshold around 63.5 could help discriminate block effectiveness and guide intraoperative decisions, although this threshold requires further validation across different surgical contexts and regional techniques.

## Abbreviations

Paravertebral Block (PVB), Analgesia Nociception Index (ANI), Instant Analgesia Nociception Index(ANIi), Mean Analgesia Nociception Index (ANIm), Local Anesthetic (LA), Magnetic Resonance Imaging (MRI), Thoracic level (T) with the number of the level, General Anesthesia (GA), Electrocardiogram (EKG), Post-Anesthesia Care Unit (PACU), Bispectral Index (BIS), Visual Analog Scale (VAS), Intra Venous (IV), Local Anesthetic Systemic Toxicity (LAST), minimal (min), 1^st^ quartile (q1), 3^rd^ quartile q3, interquartile range (iqr), standard deviation (sd), maximal (max).

## Approval

This study was approved by the French National Ethics Committee of the SUD-EST IV (Approval n° ID-RCB: 2019-A00121-56) and registered in ClinicalTrials.gov (NCT03832920). https://clinicaltrials.gov/study/NCT03832920?titles=Analgesia%20Nociception%20Index%20mastectomy&rank=2.

## Previous presentation in conferences

Abstract presented at the French Society Congress (Société Française d'Anesthésie et de Réanimation), September 19^th^, 2024 in Paris, France.

## Preprint

This trial is in a preprint process https://www.researchsquare.com/article/rs-5415736/v2?redirect=/article/rs-5415736.

## Data availability statement

The datasets generated and analyzed during the current study are not publicly available due to institutional and ethical restrictions, but de-identified data can be made available from the corresponding author upon reasonable request and with permission from the Institut de Cancérologie de Lorraine.

## Declaration of Generative AI in scientific writing

No AI was used.

## Authors’ contributions

All authors contributed to the study conception and design. Data collection were performed by JR and ASL. All authors contributed to material preparation and analysis. The first draft of the manuscript was written by JR and ASL. JR, ASL, CF and PR read and approved the final manuscript.

## Funding

This research received no specific grant from any funding agency in the public, commercial or not-for-profit sectors. Internal funding by the Institut de Cancérologie de Lorraine, Vandoeuvre-les-Nancy, France.

## Declaration of competing interest

The authors declare no conflicts of interest.
